# Exploring Associations Between Device-Based Occupational Sedentary Behavior and Need for Recovery in White Collar Workers: A Compositional Data-Analysis

**DOI:** 10.3389/ijph.2024.1607322

**Published:** 2024-07-29

**Authors:** Denise J. M. Smit, Laura J. G. C. Burgers, Sandra H. van Oostrom, Henri Vähä-Ypyä, Pauliina Husu, Simone J. J. M. Verswijveren, Karin I. Proper

**Affiliations:** ^1^Centre for Prevention, Lifestyle and Health, Department Behavior and Health, National Institute for Public Health and the Environment, Bilthoven, Netherlands; ^2^Department of Public and Occupational Health, Amsterdam Public Health Research Institute, Vrije Universiteit (VU) Medical Center, Amsterdam, Netherlands; ^3^ The UKK-Institute for Health Promotion Research, Tampere, Finland; ^4^ Institute for Physical Activity and Nutrition, School of Exercise and Nutrition Sciences, Deakin University, Geelong, VIC, Australia

**Keywords:** triaxial accelerometer, sitting, low physical workload, duration of prolonged sitting, office workers

## Abstract

**Objectives:**

White collar workers spend an increasing amount of time in occupational sedentary behavior (OSB) and are thereby at risk for adverse health outcomes. Nevertheless, the association between OSB and the need for recovery (NFR), an important indicator of wellbeing, is unknown and therefore examined.

**Methods:**

Baseline data from a cluster randomized controlled trial was used. A subgroup of 89 white collar workers wore a triaxial accelerometer for 7 days. NFR was measured using the Questionnaire on the Experience and Evaluation of Work. Compositional data analysis was applied to determine the composition of different OSB bouts (short, medium and long) and occupational physical activity (OPA) (light, moderate and vigorous and standing). Linear regression analyses were performed to explore the associations between occupational compositions and NFR.

**Results:**

Relatively more time spent in long OSB bouts was associated with a lower NFR (β: −11.30, 95% CI: −20.2 to −2.4). Short and medium OSB bouts and OPA were not associated with NFR.

**Conclusion:**

Associations between OSB bouts, OPA and NFR hinted at contrasting trends, suggesting the need to consider different bout lengths of OSB in future studies.

## Introduction

The association between physical activity (PA) and various health benefits is widely acknowledged [1, 2]. In addition to PA, attention for sedentary behavior (SB) is growing. A sedentary lifestyle is one of the key risk factors for various health problems, including diabetes, cardiovascular diseases, and all-cause mortality [3]. SB is defined as “any waking behavior characterized by an energy expenditure lower than 1.5 metabolic equivalents while in a sitting, reclining or lying posture” [4]. Besides adverse physical health outcomes, mental health outcomes, such as depression, are also linked to excessive SB [5, 6]. As there has been a shift towards more sedentary work in the past decades, attention for SB has become increasingly relevant [7]. Especially since occupational SB (OSB) is a major part of the total daily sedentary time in office-based employees. On average 60% of occupational time is spent sedentary in working adults, with up to 79% in office-based employees [8, 9]. Moreover, the duration of SB is higher on working days than on non-working days [10].

As mentioned above, both PA and SB are known to be related to physical and mental health components. In addition, high levels of self-reported SB have shown to be associated with increased fatigue and a decrease in mental wellbeing among employees [11–13]. A measure to specifically indicate work-related physical and psychological fatigue is the need for recovery (NFR) [14, 15]. The NFR is the need to recuperate from work induced efforts and the short-term workload effects after a day at work [15]. A consistently high NFR among employees is known to be associated with several health issues, including cardiovascular diseases, neck and upper limb complaints, fatigue and emotional exhaustion [15–18]. Additionally, a high NFR is associated work-related issues, such as increased absenteeism, occupational disability and early retirement [19–21]. Insight into work-related factors that lead to an increased NFR is necessary to prevent these health and work-related issues. The association between occupational PA (OPA) and NFR has been studied [22–25]. Coffeng et al. studied the association between OPA and NFR in office workers and found that, amongst others, stair climbing and (physical) detachment at work positively affected NFR. Implying that higher levels of OPA were associated with a lower NFR [22]. Two other studies indicated that increased OSB was associated with a lower NFR [24, 25]. However, the study population in these studies consisted of mainly employees with physically demanding jobs, implying that the results are specific to this occupational group [25]. Ketels et al. reported that increased OSB in physically demanding jobs attributes to the necessary breaks and subsequently leads to a decrease in the NFR, which is equivalent to an improvement [25]. However, for employees with predominantly sedentary work, higher levels of OSB might lead to unhealthy high levels of daily SB [26]. Hence, it is important to investigate the association between OSB and NFR in white collar workers, specifically.

Prolonged continuous SB of >30 min is associated with a higher risk to develop cardio-metabolic diseases, obesity or musculoskeletal disorders. On the other hand, breaks of SB, leading to shorter bouts of SB are positively associated with indicators of cardio-metabolic health [10, 27, 28]. It is therefore important to consider different bout lengths of OSB, when exploring the association between OSB and NFR in white collar workers. In doing so, it is of importance to consider the compositional nature of these different behaviors. To illustrate, a workday may consist of a closed frame of 8 h and consists of a combination of being sedentary, e.g., sitting at a desk or physically active, e.g., standing at a desk, walking. If time spent in one behavior increases then the total time spent in other behavior(s) logically decreases [29]. Thus, the time spent in one kind of movement behavior is only meaningful when the time spent in other movement behaviors is also taken into account [30]. Nevertheless, in previous studies different movement behaviors, such as physical activity and sedentary behavior, are considered to be separate variables, independent from each other [26, 31]. Although they are actually complimentary parts of a composition [31]. To incorporate different movement behaviors in a composition, compositional data analysis (CoDA) can be applied [30, 32, 33]. As this analysis considers the total combination of behaviors, e.g., all movement behaviors during a working day, instead of one single component, it is recommended to apply CoDA [30, 31, 33]. Recent studies in both the occupational and other domains already applied CoDA [24, 26, 30, 34].

Considering the above, the aim of this study was to explore the association between occupational compositions, including relative time spent in different OSB bouts and OPA, and the NFR in white collar workers, using CoDA. The research question was therefore (how) are different bout lengths of OSB and OPA associated with the NFR?

## Methods

### Study design

This study used baseline data of a subgroup of participants from the Work towards Vitality-study, a cluster-randomized controlled trial to evaluate the effectiveness of an integrated WHPP [35]. Ethical approval for the study protocol (2021.0402) was provided by the Medical Ethical Committee of the Amsterdam University Medical Center (A-UMC, Amsterdam, the Netherlands, former Medical Ethical Committee of the VUmc). The trial (NL9526) is registered in the Netherlands Trial Register. All participants provided written informed consent before participation.

### Recruitment

Participants were recruited from three organizations in different occupational sectors, i.e., two educational organizations and an assurance, tax and consulting organization. The participating organizations were recruited through the networks of the research team, co-workers and branch specific networks. Employees were recruited and informed via different communication channels, including intranet and newsletters. Additionally, all employees within the participating organizations were invited for an information session in which detailed information about the study was provided. Employees who were interested in participating in the study received and information letter, eligibility checklist and informed consent at home by post. For further details on the recruitment and details of the study design, we refer to Smit et al. [35]. For the Work towards Vitality-study, participants had to work at least 12 h per week and were excluded if they were on sick leave for more than four weeks or were pregnant. A total of 173 employees provided baseline data. For practical reasons, i.e., the availability of triaxial accelerometers, a random selection of these, i.e., the first 99 participants, were instructed to wear a triaxial accelerometer for seven consecutive days. Participants categorized as blue collar workers were excluded for the purpose of the current study (n = 7). Finally, a total of 89 participants were included, due to missing accelerometer data (n = 3).

### Data Collection

The online questionnaire including the NFR subscale of the Questionnaire on the Experience and Evaluation of Work and the accelerometer with the user instructions and diary were sent to the participants at the same moment. Some participants immediately wore the accelerometer upon completing the questionnaire, where others delayed wearing the accelerometer and/or completing the questionnaire for unknown reasons. However, both measurements took place in the same period.

#### Need for Recovery

The need for recovery (NFR) was measured using the corresponding subscale of the valid and reliable (r = 0.87) Questionnaire on the Experience and Evaluation of Work [15, 36]. Content validity was assessed by comparing NFR scores with measurement scales about fatigue at work and stress related health complaints. This showed that the NFR is a valid indicator (r = 0.65) of work-related physical and psychological fatigue [15]. The subscale comprised of 11 statements to be answered with yes or no, in which a score of 0 was assigned to the positive answer and 1 to the negative answer. An example of a statement is “Because of my job, I feel quite exhausted at the end of a working day.” The total score, i.e., the sum of the items, was standardized to a score between 0, i.e., the lowest NFR possible (most favorable score) and 100, i.e., the highest NFR possible (least favorable score).

#### Sedentary Behavior

Participants were instructed to wear a triaxial accelerometer on the hip for 24 h during seven consecutive days and to keep an activity diary to keep record of their working hours. Due to practical reasons two types of triaxial accelerometers were used to device-based measured movement behavior: the UKK RM42 and the ActiGraph GT9X Link. The UKK RM42, worn by 31 participants from organization 1, collected data within a range of ±16 g at a sampling rate of 100 Hz. The ActiGraph GT9X Link, worn by 58 participants from organization 2 and 3, had the range ±8 g at the sampling rate 30 Hz. To address differences between the sampling rate, the raw data from both types of accelerometers were processed identically, using validated mean amplitude deviation and angle for posture estimation algorithms in 6-second epochs [37]. The mean amplitude deviation algorithm describes the intensity of physical activity (PA) based on acceleration and has been found to be valid and accurate for raw triaxial accelerometer data [38]. The angle for posture estimation algorithm is responsible for measuring body posture, i.e., lying, sitting and standing and has been found to be accurate and specific [39]. The epoch-wise accelerometer output values were further smoothed by 1 min exponential moving average for each epoch time point. Therefore, short artifacts, i.e., accelerations not related to movements of interest, do not interrupt the bout calculation. In this study, only occupational time was taken into account, thus non-working days and non-working hours were excluded. The time spent in continuous OSB was split into short bouts (0–10 min), medium bouts (10–30 min) and long bouts (>30 min) of continuous OSB [10, 27, 40]. All movement behaviors other than OSB, i.e., standing, and light, moderate and vigorous PA, were considered as OPA. The bouts ending during working hours were included in the dataset. Data was classified as non-wear time if a sequence of more than 120 consecutive minutes of 0 activity was detected [41]. Non-wear time was excluded before the data was analyzed.

#### Covariates

Data about sex, age and organization was collected using an online questionnaire. The mean of the bout-based total work time was log transformed to account for the differences in working hours between participants.

### Statistical Analyses

The occupational composition, consisting of the four movement behaviors (short bouts of OSB, medium bouts of OSB, long bouts of OSB, and OPA), was transformed to a set including three isometric log ratios (ilr) [31, 33]. For the first ilr (ilr1), the first movement behavior, e.g., short OSB, was the numerator and the denominator was the geometric mean of all other movement behaviors, e.g., medium, long and OPA. The second ilr (ilr2) represented the relative time in the second movement behavior, e.g., medium OSB, *versus* the remaining movement behaviors, e.g., long and OPA, and so on (33). By rotating the sequence of the movement behaviors, each behavior was considered as the first compositional part (the numerator) once. This resulted in four ilr sets (each including three ilrs), one set for each movement behavior (Supplementary File S1). In each ilr set, the first ilr coordinate (ilr1) represented the relative importance of the first movement behavior.

To study the associations between relative time spent in each movement behavior and the NFR, four linear regression analyses were conducted, i.e., one for each movement behavior. In model 1, the ilr2 and ilr3 from the ilr set of the corresponding movement behavior and the log-transformed mean total work time were included. In model 2, three additional potential confounders, i.e., age, sex and organization, were included. All analyses were conducted in RStudio version 2023.03.1 (The R Foundation for Statistical Computing, Vienna, Austria) using the stats package and the compositions package v2.0-6 [42, 43]. The β and 95% confidence interval of each ilr1 was reported.

## Results

### Participant characteristics

Participant characteristics are presented in Table 1. The mean age of the participants was 41.4 years (SD = 11.1) and 38.2% was female. The majority of the participants (66.3%) worked partly from home, about a third worked fulltime at their workplace (30.3%) and a small proportion worked fully from home (3.4%). Most participants classified their job as low physical load (91.0%). Self-reported work hours ranged between 16 and 50 h per week with a mean of 35.4 (SD = 7.4). Self-reported working days ranged between 2 and 5 days per week with a mean of 4.4 (SD = 0.8). Outcome measures and related measures are reported in Table 2. On average, participants spent 5.1 h per day (SD = 1.3) in OSB and 2.8 h per day (SD = 1.1) in OPA. More specifically, 16.3% of the total workday was spent in short OSB, 23.5% in medium OSB, 23.3% in long OSB and 36.8% in OPA. The mean score for the NFR was 32.0 (SD = 29.7).

**TABLE 1:** Participant characteristics (Work towards Vitality study, Netherlands, 2022–2023).

Descriptive variable	Overall (N = 89)
Age in years, mean (SD)	41.4 (11.1)
Sex, female n (%)	34 (38.2%)
Work situation	
Always working at workplace n (%)	27 (30.3%)
Working from home fulltime n (%)	3 (3.4%)
Working from home, parttime n (%)	59 (66.3%)
Job intensity^a^	
Moderate physical load n (%)	3 (3.4%)
Low or light physical load n (%)	86 (96.6%)
Work hours per week, mean (SD)	35.4 (7.4)
Working days per week, mean (SD)	4.4 (0.8)

^a^ Moderate physical load: Some physical load at work, for instance occasionally lifting heavy objects. Low or light physical load: A sedentary or standing occupation, including walking but no high intensity physical activity.

**TABLE 2:** Outcome variables and related variables as measured by the triaxial accelerometers and need for recovery questionnaire (Work towards Vitality study, Netherlands, 2022–2023).

Outcome measure	Result
OSB hours per day, mean (SD)	5.1 (1.3)
OPA hours per day, mean (SD)	2.7 (1.1)
Short OSB bouts, mean % of the total workday^a^	16.3%
Medium OSB bouts, mean % of the total workday^b^	23.6%
Long OSB bouts, mean % of the total workday^c^	23.3%
OPA bouts, mean % of the total workday	36.8%
Need for recovery, mean, (SD)^d^	32.0 (29.7)

Abbreviations: OSB, Occupational sedentary behavior; OPA, Occupational physical activity.

^a^ Bouts of 0–10 min of OSB.

^b^ Bouts of 10–30 min of OSB.

^c^ Bouts of >30 min of OSB.

^d^ NFR, ranges from 0 to 100.

### Occupational compositions and the need for recovery

Results from model 1 (β = −12.2, 95% CI = −21.7–−2.7) and model 2 (β = −11.3, 95% CI = −20.2–−2.4) indicate that more time spent in long OSB bouts, relative to short-, medium- and OPA bouts was associated with a lower need for recovery (Table 3). Albeit the three other associations, i.e., short and medium OSB and OPA with NFR, were not statistically significantly associated, trends in different directions for each variable were apparent. We observed a negative effect size suggesting lower NFR when more time was spent in short OSB bouts relative to medium-, long- and OPA bouts according to both model 1 (β = −11.0, 95% CI = −29.7– 7.7) and 2 (β = −18.3, 95% CI = −37.1 – 0.5). Further, for more time spent in medium OSB bouts (model 1: β = 8.9, 95% CI = −11.1 – 28.9, model 2: β = 16.2, 95% CI = −3.0 – 35.4) and OPA (model 1: β = 14.2, 95% CI = −3.8 – 28.9, model 2: β = 13.4, 95% CI = −5.0 – 31.8) relative to the other bouts, positive effect sizes, though not statistically significant, were apparent indicating a higher NFR.

**TABLE 3:** Effect estimates and 95% confidence intervals of the four linear regression analyses for the associations between irl1 (from each movement behavior) and the need for recovery (Work towards Vitality study, Netherlands, 2022–2023).

Ilr1 of the first movement behavior	Model 1^a^		Model 2^b^	
	β	95% CI	β	95% CI
Short OSB bouts	−11.0	−29.7–7.7	−18.3	−37.1–0.5
Medium OSB bouts	8.9	−11.1–28.9	16.2	−3.0–35.4
Long OSB bouts	−12.2	−21.7 to −2.7*	−11.3	−20.2 to −2.4*
OPA bouts	14.2	−3.8–32.2	13.4	−5.0–31.8

Abbreviations: OSB, Occupational sedentary behavior; OPA, Occupational physical activity.

*Significant association.

^a^ Model 1 adjusted for ilr2, ilr3, and the log transformed mean worktime.

^b^ Model 2 additionally adjusted for age, sex, and organization.

## Discussion

The aim of the current study was to explore the associations between relative time spent in different OSB bouts and OPA and the need for recovery in white collar workers. Results indicate an association between more time spent in long OSB bouts relative to the other OSB bouts and OPA and a lower need for recovery. There was a negative effect size, though not statistically significant, suggesting a lower need for recovery, when more time was spent in short OSB bouts relative to the other OSB bouts and OPA. On the other hand, for more time spent in both medium OSB bouts and OPA relative to other OSB bouts, positive effect sizes though not statistically significant, indicating a higher need for recovery were observed.

Two previous studies investigated associations between different occupational movement behaviors, including OSB, and NFR, of which one study also applied CoDA [24, 25]. Stevens et al. included occupational SB, standing, light PA, and moderate to vigorous PA in their compositions [24]. Ketels et al. studied the associations between occupational SB, standing and moderate to vigorous PA and NFR [25]. Both studies indicated that more time spent in overall OSB compared to the other occupational movement behaviors resulted in a lower need for recovery [24, 26]. This finding is in line with results from our study. However, two notable differences between these studies and our study should be considered. First of all, both Stevens et al. and Ketels et al. did not make a distinction between different bout lengths of OSB [24, 25]. Secondly, the study populations differed from our study. Stevens et al. included predominantly employees with physically demanding jobs and a small proportion of white-collar workers, i.e., administration workers [24] and Ketels et al. only included employees with physically demanding jobs [25].

In our study, the results pointed in different directions for the different bout lengths. This indicates that the different bout lengths should be considered in future studies exploring OSB. Furthermore, the bout lengths can also be specified further. We focused on bout lengths of 0–10 min, 10–30 min and those exceeding 30 min of OSB, as detrimental health outcomes are associated with prolonged sitting of over 30 min [10, 28]. However, in potential there might also be a different association with NFR for sitting continuously for 30–60 min and bouts longer than 60 min. In our study, we concentrated solely on participants classified as white-collar workers. In future studies that further explore the associations between OSB and NFR, it is important to capture a broader range of occupational contexts and consider the specific occupations that vary in (physical) work demands. For instance, the workday of a teacher might differ substantially from the workday of an office worker with regard to OSB and OPA.

As prolonged SB is known to be a risk factor for, amongst others, diabetes and cardiovascular diseases, the finding that more time spent in long OSB bouts relative to the other OSB bouts and other movement behaviors was associated with a lower need for recovery might be unexpected [3]. Another striking observation was that effect sizes in different directions were apparent for the different OSB bouts and OPA. The impact of task interruption might be a possible explanation for the observed trends in opposite directions. According to Mark et al., it takes approximately 25 min to resume a task after an interruption [44]. Interruptions cause an increase of task completion time and a decrease of task performance [45]. Which in turn can lead to unfinished work and tasks at the end of the day. Unfinished work or tasks are identified as a job-related stressor and may result in diminished detachment from work which subsequently might lead to a higher need for recovery [46, 47]. Long OSB bouts might imply less interruptions from work tasks and thus a lower need for recovery after work. However, medium OSB bouts indicate that the participants interrupted sitting within 30 min, and potentially also their task. This might be attributed to increased task completion time and unfinished work at the end of the day, resulting in a higher need for recovery. Although formulating concrete recommendations for practice based on the results of an exploratory study might be too early, existing literature supports dynamic workplaces such as desk bikes or sit/stand stations that allow posture changes and could thus interrupt OSB, without interrupting cognitive work and productivity [48–50].

As cross-sectional data was used for this study, it cannot be assumed that the association between medium OSB bouts and a higher need for recovery is causal. The possibility that a high NFR affected the OSB bouts should therefore be considered. For instance, an employee with a high need for recovery might have difficulties with regaining concentration and completing tasks. If they also interrupt OSB when they are not able to finish a task, this can lead to more medium OSB bouts [15]. Longitudinal studies using accelerometry are required to gain more insight in the causal relationship between different OSB bouts and NFR as well as underlying mechanisms. In our study, the activities and tasks conducted during a workday throughout the accelerometry measurement period were not reported, but provide more insight in the association between OSB bouts and the need for recovery. Hence, more detailed information about the tasks throughout a workday should be gathered in future studies.

OPA bouts included standing and different intensities of physical activity. Potentially, results might have been different if standing, light PA and moderate to vigorous PA were considered as separate movement behaviors, which is also reported in another study [25]. Higher levels of OPA could indicate employees are attending a large amount of appointments that require them to walk to another location. A high number of consecutive appointments on a day may induce fatigue or stress, potentially affecting the NFR. A larger amount of OPA could also indicate more breaks for both OSB and completing tasks. This could attribute to the observed higher need for recovery associated with OPA bouts.

A strength of the study is the device-based movement behavior during occupational time, which is more reliable than subjectively measured movement behavior by questionnaires or activity diaries [51]. Another strength of this study is the application of CoDA and including different bout lengths of OSB. As it accounts for the codependency between movement behaviors, enhancing the robustness of the findings.

Two types of accelerometers were used, which may be considered a limitation. However, discrepancies in the sampling rate (100 Hz vs. 30 Hz) were addressed by using the same algorithms which are proven to enable comparison of accelerometer data irrespective of accelerometer features such as sampling rate, range and resolution [37]. Another limitation is the small sample size included (n = 89). A *post hoc* power analysis, based on an *R* ^2^ of 0.1, 7 determinants and an alpha of 0.05, revealed that the power of the current study was 56%. Given this power, it might not have been feasible to detect significant associations. Studies including a larger sample and thus greater power, are necessary to gain more insight in the associations between OSB bouts, OPA and NFR. Lastly, the working hours reported in the diary and derived from the accelerometer were not fully synchronized. All bouts that ended during a working day were included. However, it could occur that a participant was in a certain movement behavior and ended the working day, i.e., time reported in the diary, but remained in this movement behavior after the working day ended. This bout was excluded as it did not end during the working day. An average difference of 5 min between working time reported in the diaries and the sum of OSB bouts and OPA was observed and was not expected to affect the outcomes, as 5 min is only a small part of the average workday (7.8 h).

### Conclusion

In conclusion, our study revealed that long bouts of OSB relative to the other OSB bouts and OPA were associated with a lower NFR. This suggests that extended OSB bouts may indicate fewer interruptions from work tasks, subsequently reducing the necessity for post-work recovery. Hence, it is important to assess the effect of interventions, such as dynamic workplace solutions, on OSB bouts, OPA and NFR. Additionally, results from this study imply the need to consider different bout lengths of OSB. To gain insight in the causal relation between different bout lengths of OSB and NFR and the role of the occupational setting and work tasks, longitudinal studies with larger sample sizes are required.

## Supplementary Material

SUPPLEMENTARY FILE S1 Formulas to calculate ilr coordinates (ilr1, ilr2, ilr3) for each movement behavior, i.e. short, medium and long bouts of occupational sedentary behavior and occupational physical activity (Work towards Vitality study, Netherlands, 2022–2023).
