# Supplementary material for: Exploring Associations Between Device-Based Occupational Sedentary Behavior and Need for Recovery in White Collar Workers: A Compositional Data-Analysis
Source: Int J Public Health. 2024 Jul 29;69:1607322. doi: 10.3389/ijph.2024.1607322 (PMC11317241; doi:10.3389/ijph.2024.1607322)
Supplement: Supplementary file 1 [file DataSheet1.pdf]

International Journal of Public Health

Exploring Associations Between Device-Based Occupational Sedentary Behavior and Need for Recovery in White Collar Workers: A Compositional Data-Analysis

Supplementary file 1. Formulas to calculate ilr coordinates (ilr1, ilr2, ilr3) for each movement behavior, i.e. short, medium and long bouts of occupational sedentary behavior and occupational physical activity (Work towards Vitality study, Netherlands, 2022-2023).

Ilr set for short OSB bouts[a] :

$$ilr_{1OSBs} = \sqrt{\frac{3}{4}}\ln\left(\frac{OSBshort}{\sqrt[3]{OSBmedium * OSBlong * OPA}}\right)$$

$$ilr_{2OSBs} = \sqrt{\frac{2}{3}}\ln\left(\frac{OSBmedium}{\sqrt[2]{OSBlong * OPA}}\right)$$

$$ilr_{3OSBs} = \sqrt{\frac{1}{2}}\ln\left(\frac{OSBlong}{OPA}\right)$$

Ilr set for medium OSB bouts[b] :

$$ilr_{1OSBm} = \sqrt{\frac{3}{4}}\ln\left(\frac{OSBmedium}{\sqrt[3]{OSBshort * OSBlong * OPA}}\right)$$

$$ilr_{2OSBm} = \sqrt{\frac{2}{3}}\ln\left(\frac{OSBshort}{\sqrt[2]{OSBlong * OPA}}\right)$$

$$ilr_{3OSBm} = \sqrt{\frac{1}{2}}\ln\left(\frac{OSBlong}{OPA}\right)$$

Ilr set for long OSB bouts[c] :

$$ilr_{1OSBl} = \sqrt{\frac{3}{4}}\ln\left(\frac{OSBlong}{\sqrt[3]{OSBshort * OSBmedium * OPA}}\right)$$

$$ilr_{2OSBl} = \sqrt{\frac{2}{3}}\ln\left(\frac{OSBshort}{\sqrt[2]{OSBmedium * OPA}}\right)$$

$$ilr_{3OSBl} = \sqrt{\frac{1}{2}}\ln\left(\frac{OSBmedium}{OPA}\right)$$

Ilr set for OPA bouts[d] :

$$ilr_{1OPA} = \sqrt{\frac{3}{4}} \ln\left(\frac{OPA}{\sqrt[3]{OSBshort * OSBmedium * OSBlong}}\right)$$

$$ilr_{2OPA} = \sqrt{\frac{2}{3}} \ln\left(\frac{OSBshort}{\sqrt[2]{OSBmedium * OSBlong}}\right)$$

$$ilr_{3OPA} = \sqrt{\frac{1}{2}} \ln\left(\frac{OSBmedium}{OSBlong}\right)$$

[a] Short bouts of occupational sedentary behavior (0-10 minutes). [b] Medium bouts of occupational sedentary behavior (10-30 minutes). [c] Long bouts of occupational sedentary behavior (>30 minutes). [d] Occupational physical activity (standing, walking and light, moderate and vigorous physical activity).
